# Serum-Induced Expression of Brain Natriuretic Peptide Contributes to Its Increase in Patients with HFpEF

**DOI:** 10.3390/ijms23062991

**Published:** 2022-03-10

**Authors:** Ryuji Okamoto, Ryotaro Hashizume, Noboru Suzuki, Rie Ito, Tomoko Tokuhara, Hiroshi Fujiwara, Ye Zhe, Hiromasa Ito, Takaya Abe, Kaoru Dohi

**Affiliations:** 1 Department of Cardiology and Nephrology, Mie University Graduate School of Medicine, Tsu 514-8507, Japan; itoyuriri@yahoo.co.jp (R.I.); ye.zhe@outlook.com (Y.Z.); h-ito@clin.medic.mie-u.ac.jp (H.I.); dohik@clin.medic.mie-u.ac.jp (K.D.); 2 Regional Medical Support Center, Mie University Hospital, Tsu 514-8507, Japan; 3 Department of Pathology and Matrix Biology, Mie University Graduate School of Medicine, Tsu 514-8507, Japan; hashizumer@doc.medic.mie-u.ac.jp; 4 Department of Animal Genomics, Functional Genomics Institute, Mie University Life Science Research Center, Tsu 514-8507, Japan; nsuzuki@doc.medic.mie-u.ac.jp; 5 Laboratory for Animal Resources and Genetic Engineering, RIKEN Center for Biosystems Dynamics Research, Kobe 650-0047, Japan; tomoko.tokuhara@riken.jp (T.T.); takaya.abe@riken.jp (T.A.); 6 Department of Personalized Cancer Immunotherapy, Mie University Graduate School of Medicine, Tsu 514-8507, Japan; hi-fuji@pd6.so-net.ne.jp

**Keywords:** agonist, brain natriuretic peptide, cardiomyocyte, heart failure, patient’s serum

## Abstract

Brain natriuretic peptide (BNP) levels are increased in both patients with heart failure with preserved (HFpEF) and reduced ejection fraction (HFrEF), but the reasons for this remain unclear. Our purpose was to examine whether serum-induced BNP (iBNP) expression partly contributes to increased BNP in patients with HFpEF. BNP reporter cardiomyocytes from pBNP-luc-KI mice were stimulated with serum from patients with HFpEF or HFrEF (*n* = 114 and *n* = 82, respectively). Luciferase activity was examined as iBNP and the iBNP-to-BNP ratio was evaluated. Patient characteristics and clinical parameters were compared, and multivariate regression analysis was performed to determine independent predictors of the iBNP-to-BNP ratio. Female sex and frequencies of atrial fibrillation, hypertension and the use of a calcium channel blocker (CCB) were higher in HFpEF. The iBNP-to-BNP ratio was significantly higher in HFpEF (26.9) than in HFrEF (16.1, *p* < 0.001). Multivariate regression analysis identified the existence of HFpEF as an independent predictor of the iBNP-to-BNP ratio after adjusting for all other measurements (β = 0.154, *p* = 0.032). Age, hemoglobin, CCB usage and deceleration time were also independent predictors (β = 0.167, *p* = 0.025; β = 0.203, *p* = 0.006; β = 0.138, *p* = 0.049; and β = 0.143, *p* = 0.049, respectively). These results indicate that the elevated BNP in patients with HFpEF is partly due to iBNP from the heart.

## 1. Introduction

The number of patients with heart failure with preserved ejection fraction (HFpEF) is increasing, representing a growing burden as a health problem around the world [1,2]. Prognosis is poor for patients with HFpEF, similar to that of patients with HF with reduced ejection fraction (HFrEF) [1,3]. Neurohormonal antagonists, including angiotensin-converting enzyme inhibitor (ACEi)/angiotensin II receptor blocker (ARB), mineralocorticoid receptor antagonists (MRA), beta-blockers and angiotensin receptor neprilysin inhibitors (ARNI) have been shown to improve survival and hospitalization in patients with HFrEF. However, all these agents have failed to improve mortality in patients with HFpEF [3,4,5]. The trials of sodium/glucose cotransporter 2 inhibitors (SGLT2i) for the treatment of HFpEF are ongoing [6]. Thus, pathological mechanisms underlying HFpEF should be clarified and new agents and/or devices to improve the prognosis of HFpEF developed [7].

Brain natriuretic peptide (BNP) belongs to a family of vasoactive peptide hormones with favorable physiological properties [8]. BNP is an established diagnostic biomarker for both HFpEF and HFrEF [9], and also for cardiac hypertensive hypertrophy [10]. The biological effects of BNP include vasorelaxation, natriuresis, and diuresis, leading to the regulation of blood pressure and body fluid volume [11,12]. However, how BNP is regulated remains unclear, especially in terms of upstream signaling. A stretch receptor that has not yet been fully identified is believed to stimulate the expression of BNP in proportion to ventricular wall stress [13]. Candidates for undetermined stretch-activated receptors include mechanically gated channels, which can be activated by mechanical stimuli alone, and mechanically modulated channels, which require nonmechanical stimuli such as agonists [14]. Serum-induced expression of BNP may thus contribute to increased BNP in HF patients independent of hemodynamic effects. We hypothesized that some levels of BNP are induced by agonists in serum, in parallel with cardiac wall stress. We developed a system to measure serum-induced BNP (iBNP) expression using knock-in (KI) technology, then compared effects between HFpEF and HFrEF patients. We incubated cardiomyocytes from pBNP-luc-KI mice with serum from patients and measured luciferase (luc) activity as iBNP.

## 2. Results

### 2.1. Patient Characteristics

In this study, 196 patients with HF were recruited (Table 1). Table 1 shows the characteristics of patients in the HFpEF and HFrEF groups. Mean ages were 67 and 71 years in the HFrEF and HFpEF groups, respectively. Significant differences between groups were observed in sex, systolic and diastolic blood pressures, heart rate, LVEF, BNP, number of hospitalizations for HF during the past 12 months, and prevalences of atrial fibrillation, coronary artery disease, hypertension, chronic kidney disease, diabetes mellitus, ever-smoker status, and uses of medications including ACEi/ARB, beta-blocker, calcium channel blockers (CCB), MRA and diuretic. In addition, RV5 + SV1 from electrocardiography, interventricular septal wall thickness, posterior wall thickness, LVEF, A wave, and deceleration time of mitral E-wave velocity from echocardiography were significantly higher in the HFpEF group than in the HFrEF group. Left atrial dimension, left ventricular end-diastolic and end-systolic dimensions, left ventricular mass index, and the E-to-E’ ratio were significantly lower in HFpEF. Laboratory examinations showed Na, Cl, high-density lipoprotein, and estimated glomerular filtration rate were significantly higher in HFpEF than in HFrEF (Table 1).

### 2.2. Development of a BNP Reporter Mouse and Investigation of Patient iBNP Expression

**Figure 1:**
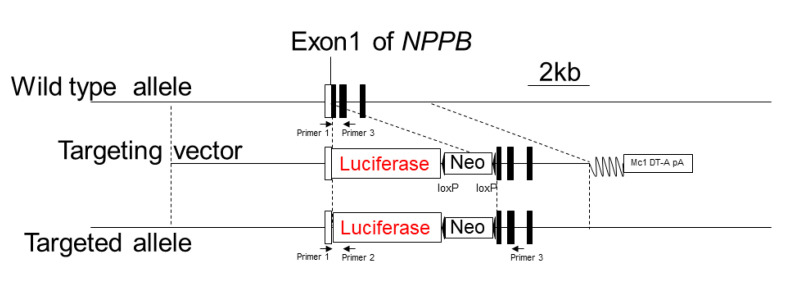
Targeting strategy by homologous recombination. Structures of the wild-type allele (**top** row), gene targeting vector (**middle row**) and targeted allele (**bottom row**) are shown. DT-A, diphtheria toxin-A cassette; Neo, neomycin cassette; *NPPB*, natriuretic peptide B gene.

**Figure 2:**
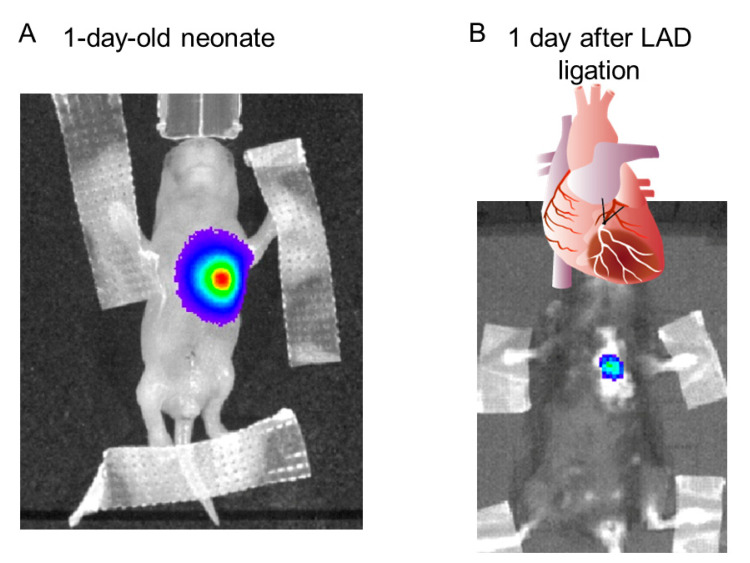
(**A**) Detection of luciferase activity in one-day BNP reporter mouse. (**B**) Detection of luciferase activity in reporter mouse one day after ligation of the left anterior descending coronary artery (LAD). BNP, brain natriuretic peptide.

**Figure 3:**
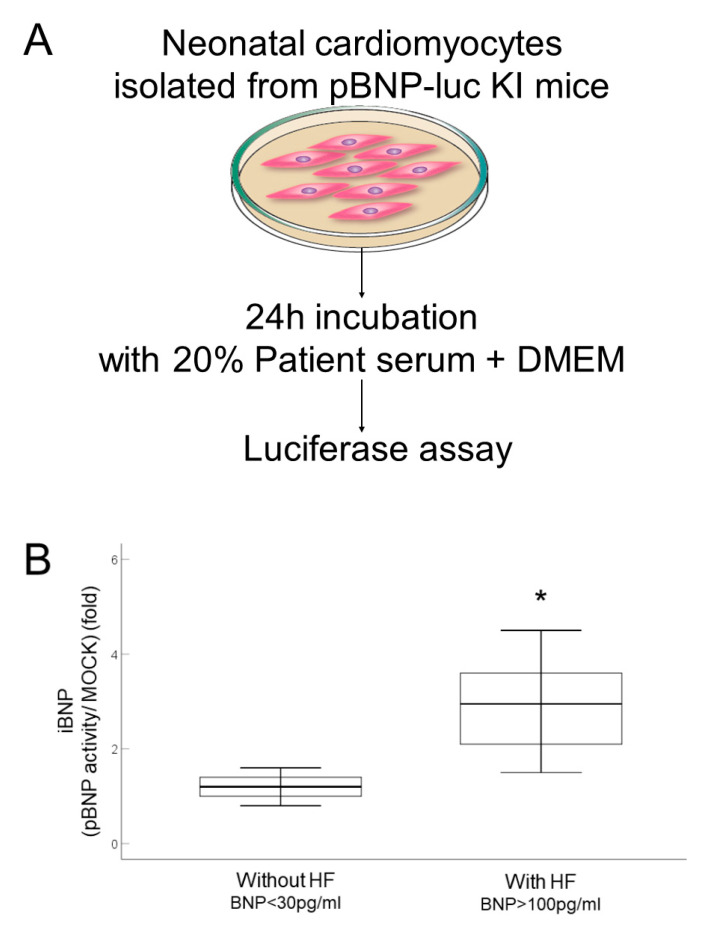
The BNP reporter system and activation of the BNP promoter in cardiomyocytes treated with serum from patients with heart failure. (**A**) Assay flow to measure serum-induced BNP (iBNP) expression. pBNP-luc KI mice, luciferase knock-in (KI) mice with site-specific integration of luciferase gene, which is regulated by, and acts as, an endogenous promoter of BNP (pBNP). DMEM, Dulbecco’s modified Eagle’s medium. (**B**) Box plot showing iBNP in cardiomyocytes from pBNP-luc KI mice stimulated with 20% serum from patients without, and with, heart failure (HF) for 24 h. *n* = 10. * *p* < 0.05 vs. without HF. BNP, brain natriuretic peptide; DMEM, Dulbecco’s Modified Eagle Medium.

To generate the BNP reporter mice (pBNP-luc-KI mice), the luciferase cDNA was inserted into the initiation site of the *BNP* gene, *NPPB* (Figure 1). In vivo imaging of luciferase showed reactivation of BNP in adult mice after ligation of the left anterior descending coronary artery, as a model of human adult heart disease (Figure 2). Next, we isolated cardiomyocytes from pBNP-luc KI mice and observed activation of the BNP promoter after stimulation with 1 μM of angiotensin II (data not shown). In this system, we tried to stimulate reporter cardiomyocytes with serum from patients with HF (Figure 3A). Interestingly, iBNP expression was significantly higher in patients with HF (BNP > 100 pg/mL) than in patients without HF (BNP < 30 pg/mL) (Figure 3B).

### 2.3. The iBNP-to-BNP Ratio Was Significantly Higher in HFpEF Patients Than in HFrEF Patients

**Figure 4:**
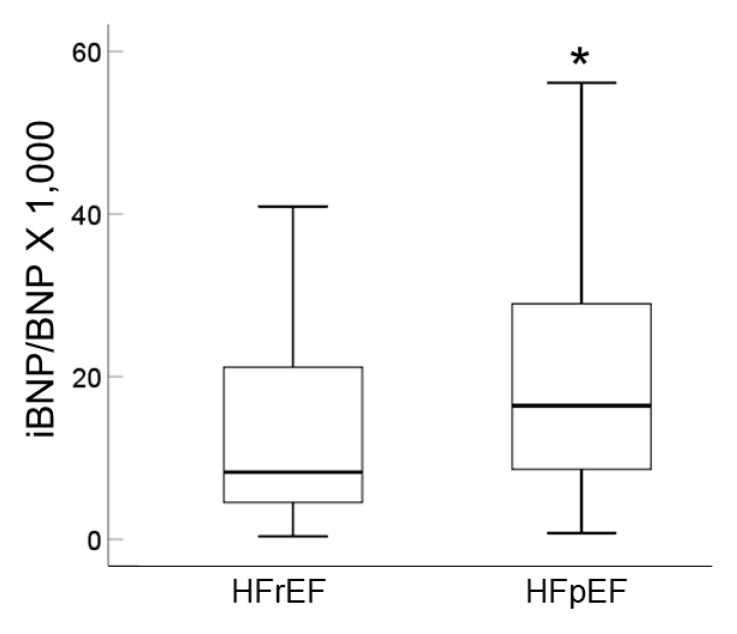
Box plot of the iBNP-to-BNP ratio in HFpEF and HFrEF. HF, heart failure; BNP, brain natriuretic peptide; iBNP, serum-induced BNP expression; pEF, preserved ejection fraction; rEF, reduced ejection fraction. * *p* < 0.05 vs. HFrEF.

Although BNP levels are well-known to be lower in HFpEF than in HFrEF [15], we examined the effect of serum from HFpEF patients on cardiomyocytes from pBNP-luc KI. The iBNP-to-BNP ratio was significantly increased in cardiomyocytes after stimulation with serum from HFpEF patients compared to that from HFrEF patients (Figure 4). Multivariate regression analysis identified HFpEF, age, hemoglobin, use of CCBs and deceleration time as independent predictors of the iBNP-to-BNP ratio (Table 2). These results suggest that iBNP partly contributes to the elevation of BNP in patients with HFpEF.

## 3. Discussion

Many investigators have tried to clarify differences between HFpEF and HFrEF, including predictors of onset [16], comorbidities [17], modes of death [18,19], drug response [20,21,22], metabolic changes [23,24], biomarkers [25], microRNA [26,27], and exercise intolerance [28]. However, definitive therapies to improve mortality and/or HF hospitalization in patients with HFpEF are still lacking. In this study, we noticed increased activation of the BNP promotor by serum from HFpEF patients compared with HFrEF. BNP is well known to be induced by cardiomyocytes after agonist stimulation without stretch [29]. Therefore, iBNP would naturally be expected to contribute to this increase in patients with HF to some degree. We speculate that undetermined stretch-activated receptors to produce BNP might be mechanically modulated channels, which require nonmechanical stimuli as agonists [14], because the elevation of BNP was partly due to iBNP. It remains unclear, however, which substances in serum stimulate pBNP, especially in HFpEF. Metabolomic differences in serum may exist between patients with HFpEF and HFrEF, according to a Canadian registry study [24]. This team also proposed that an inflammatory response via tumor necrosis factor-alpha (TNFα) could play an important role in the development of HFpEF [30]. A network analysis recently showed biomarker interactions mostly related to inflammatory pathway signaling (TNFα, suppression of tumorigenicity 2 (ST2), vascular endothelial growth receptor (VEGF), etc.) in patients with HFpEF, whereas biomarkers were mostly related to cardiac stretch in HFrEF patients [31]. Inflammatory responses can thus play important roles in the development of HFpEF and elevation of biomarkers in HFpEF patients compared with HFrEF [32]. Indeed, inflammation can induce gene expression and secretion of BNP [33]. Further investigation is necessary to identify those substances that activate pBNP and determine whether they can be inhibited by anti-inflammatory drugs such as anti-interleukin (IL)-1β antibody [34], statins, etc. [22].

**Figure 5:**
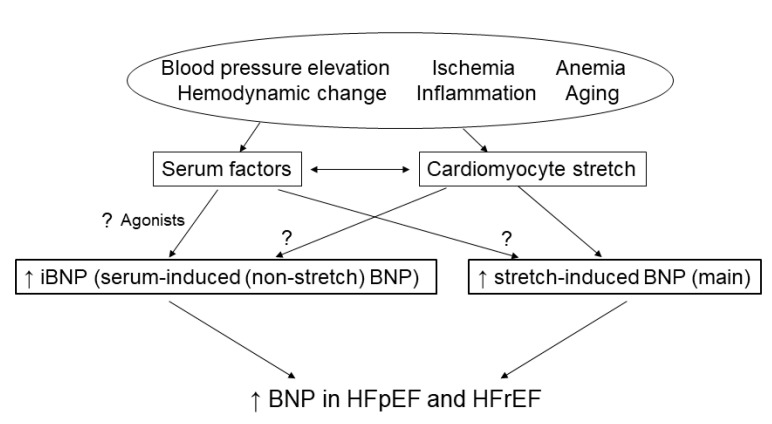
Possible mechanism of iBNP elevation in HF patients. BNP, brain natriuretic peptide; HF, heart failure; iBNP, serum-induced BNP expression; pEF, preserved ejection fraction; rEF, reduced ejection fraction. ? means it remains unknow. ↑ means increased expression.

We identified the existence of HFpEF as an independent predictor of the iBNP-to-BNP ratio, in addition to age, hemoglobin, use of CCBs, and deceleration time. The finding that age is an independent predictor of the iBNP-to-BNP ratio is interesting. Indeed, elderly individuals are well-known to show higher BNP concentrations even in the absence of cardiac disease [15]. Hemoglobin was also related to the iBNP-to-BNP ratio. This may be attributable in part to the fact that anemia easily contributes to increases in BNP via hemodynamic effects, especially in HFrEF. The use of a CCB means the patient is more hypertensive. Indeed, blood pressure was higher in HFpEF than in HFrEF (Table 1). The effects of hypertension on cardiomyocytes and cardiac structures can lead to the elevation of iBNP (Figure 5). The deceleration time of mitral inflow E velocity is a well-known marker of diastolic dysfunction [35]. This time is prolonged in patients with a relaxation abnormality such as the predominant diastolic dysfunction, because left atrial and left ventricular pressures take longer to equilibrate, with a slower and continued decrease in left ventricular pressure until mid-to-late diastole. The iBNP-to-BNP ratio may be related to diastolic dysfunction.

Recently, ARNI have been used as a first-line therapy for patients with HF. ARNI play an important role in the treatment of HF by raising endogenous natriuretic peptides including BNP [36]. It might be interesting to see if ANRI can raise more BNP, especially derived from iBNP. Further investigations are necessary to clarify the role of iBNP-to-BNP ratio in HFpEF and HFrEF (Figure 5).

Obesity and diabetes mellitus have also been reported to contribute to systemic inflammation, leading to cardiac remodeling and the development of HFpEF [37,38]. Although body mass index and the prevalence of diabetes mellitus were higher in HFpEF than in HFrEF (Table 1), these were not recognized as independent predictors of the iBNP-to-BNP ratio (Table 2). Further investigations are necessary to reveal the correct mechanisms of iBNP elevation.

In summary, we have developed a system to measure iBNP using KI technology. This system showed that the iBNP-to-BNP ratio was significantly higher in HFpEF than in HFrEF, suggesting that iBNP may play an important role in the development of HFpEF and may help clarify the pathophysiological differences between HFpEF and HFrEF.

## 4. Materials and Methods

### 4.1. Animal Preparation

All protocols were approved by the Institutional Animal Care and Use Committee of Mie University (protocol no. 24–25) and RIKEN Kobe Branch (Approval number: A2001-03).

### 4.2. Development of BNP Reporter Mice

To generate the BNP in reporter mice (Accession. No. CDB1373K: http://www.clst.riken.jp/arg/mutant%20mice%20list.html, accessed on 7 December 2021), the luciferase cDNA was inserted into the initiation site of the mouse BNP gene (*NPPB*, GenBank #NC_000070.6, accessed 22 July 2017) A targeting vector was constructed to replace the coding region of exon 1 of *NPPB* with a DNA fragment containing a luciferase cDNA and loxP-flanked neomycin resistance gene driven by PGK promoter (Figure 1). A diphtheria toxin A fragment gene driven by the MC-1 promoter at the 3’ end of the targeting vector was used for negative selection. The linearized targeting vector was introduced into the HK3i C57BL/6N embryonic stem (ES) cells [39], and cells were selected in G418. Genomic DNA from ES cell clones was screened by PCR. Homologous recombination was confirmed by Southern blot analysis using external 3’ and 5’ and neomycin probes. A correctly targeted ES clone was injected into 8-cell stage embryos. Chimeric mice were mated with C57BL/6 female mice for germinal transmission. Heterozygous mice (pBNP-Luc knock-in (KI)) were backcrossed to C57BL/6J for at least seven generations. PCR genotyping was performed using the following primers: Primer 1, CCGGCAGGAATGCAGCTGATAAATC; Primer 2, GGCCATGTTGTTGTCCTCGGAGGA; Primer 3, GATGGGATGGGTGTGCAGCTTTGC. PCR product sizes are 1000 and 779 base pairs for knock-in and wild-type alleles, respectively. The pBNP-luc-KI mouse is available (R. Okamoto: ryuji@clin.medic.mie-u.ac.jp).

### 4.3. Assessment of NPPB Promotor Activation by CCD Camera/IVIS Imaging

Mice received 125 mg/kg of luciferin substrate by intraperitoneal injection. Image acquisition was performed on anesthetized mice receiving isoflurane (2%). Bioluminescence signals were collected using an in vivo imaging system (IVIS Lumina 2; Caliper Life Science, Cheshire, UK). Light intensity of bioluminescence was recorded as maximum photon counts (radiance, photons/s/cm^2^/steradian) within a region of interest (ROI). Serial images were collected from the heart in the ventral view for 40 min after luciferin injection in 5-min intervals.

### 4.4. Myocardial Infarction in Mice

Myocardial infarction (MI) by ligation of the left anterior descending coronary artery was studied in 10 to 12-week-old male C57BL/6J mice (SLC), as described previously [40]. Anesthesia was induced with 3.0% isoflurane inhalation with 100% oxygen, followed by intubation and respiratory support with a rodent volume-controlled mechanical ventilator (VentElite 55-7040; Harvard Apparatus, Holliston, MA, USA) at a tidal volume of 3 mL and a respiratory rate of 80 breaths/min. Fourth left thoracotomy was performed to expose the heart, followed by ligating the proximal left anterior descending coronary artery with a 12-0 polypropylene suture. Myocardial ischemia was confirmed as decreased movement in the free wall of the left ventricle and regional cyanosis.

### 4.5. Serum-Induced under Promotor of BNP (pBNP) Luciferase Analysis

Mouse neonatal cardiomyocytes were isolated from the ventricles of 2-day-old pBNP-luc-KI mice and luciferase (luc) knock-in (KI) mice with site-specific integration of the luciferase gene, which is regulated by, and acts as an endogenous promotor of, BNP (pBNP), as described previously [41]. Cells were incubated for 24 h with 0.1 mL of medium, comprising 20% fetal bovine serum (FBS) (negative control, MOCK), 20% patient-derived serum or 20% FBS and 1 μM angiotensin II (positive control, Peptide Institute 4001; Osaka, Japan) in phenol red-free Dulbecco’s modified Eagle medium (Invitrogen 21063-029; Carlsbad, CA, USA) and penicillin-streptomycin in fibronectin-coated 96-well plates (Figure 1). Cells were observed using a fluorescent microscope (BZ-X710; Keyence, Osaka, Japan). Cells were then washed with phosphate-buffered saline three times, a 1 × lysis reagent was dispensed, mixed with a luciferase assay agent and analyzed for luciferase activity according to the protocol from the manufacturer (Luciferase Assay System, E1500; Promega, Fitchburg, WI, USA). Luciferase activity was measured by a plate reader (ARVO X2; PerkinElmer, Waltham, MA, USA). We defined iBNP as pBNP-luc activity with serum/pBNP-luc activity in FBS (MOCK).

### 4.6. Study Population

The study protocol was approved by the Ethics Committee at Mie University Hospital (reference number: 2893). This study was performed using an opt-out methodology. The opt-out option was presented on the hospital’s website and as a notice in a prominent place at Mie University Hospital.

### 4.7. Diagnosis of HFpEF and HFrEF

HF was defined using a combination of signs and symptoms, as previously reported [2]. We divided patients into HFpEF and HFrEF groups based on left ventricular ejection fraction (LVEF) measured from echocardiography at the time of enrollment. Patients with LVEF > 50% were classified as having HFpEF and those with LVEF < 40% were classified as HFrEF on standard pharmacotherapy. We excluded patients with mid-range HF(LVEF 40–50%), residual coronary artery disease needing revascularization and patients with HF mainly due to valvular heart disease and/or congenital structural disease. Hypertensive patients with normal BNP concentration and no history of HF were also included in this study.

### 4.8. Statistics

Data are reported as mean ± standard deviation. The chi-squared test or Mann–Whitney test was used to compare baseline characteristics. Multivariate regression analyses were performed to identify factors from among risk factors and laboratory data that were associated with the ratio of iBNP to BNP (iBNP-to-BNP ratio). Values of *p* < 0.05 were considered significant. Data were processed using SPSS version 25 software (IBM, Chicago, IL, USA).

## 5. Conclusions

These results indicate that the elevated BNP in patients with HFpEF is partly due to iBNP from the heart.

## 6. Limitations

Our recruited patients did not receive angiotensin receptor neprilysin inhibitor (ARNI) or sodium/glucose cotransporter 2 inhibitor (SGLT2i), which are important drugs in the latest guideline [36], because they were not available for the treatment of heart failure in Japan until August and December 2020, respectively. We did not evaluate high-sensitivity CRP, IL-1, IL-6, ST2, TNFα, or VEGF, all of which are considered inflammatory regulators in cardiovascular diseases. Further investigation is necessary to evaluate the roles and mechanisms of iBNP elevation in HF.

## Figures and Tables

**Table 1:** Patient characteristics.

Variables	All, *n* = 196	HFrEF, *n* = 82	HFpEF, *n* = 114	*p* Value
Age, y	69 ± 14	67 ± 15	71 ± 14	0.14
Male gender, *n* (%)	118 (60)	56 (68)	62 (54)	0.007
Body Mass Index, kg/m^2^	23.5 ± 3.9	22.8 ± 4.2	23.9 ± 3.6	0.067
Systolic blood pressure, mmHg	138 ± 17	114 ± 26	133 ± 26	7.1 × 10^−8^
Diastolic blood pressure, mmHg	84 ± 11	68 ± 14	74 ± 15	0.006
Heart rate in ECG, beats/min	72 ± 16	77 ± 16	69 ± 15	1.9 × 10^−4^
BNP (pg/mL)	552.4 ± 1190.7	899.9 ± 1766.4	302.6 ± 244.0	6.6 × 10^−7^
No of hospitalizations for heart failure during the past 12 months (%)	43 (42)	77 (94)	65 (57)	1.2 × 10^−8^
Comorbidities, *n* (%)				
Atrial Fibrillation	49 (26)	14 (17)	35 (31)	1.9 × 10^−7^
Coronary artery disease	58 (30)	34 (42)	24 (21)	0.002
Hypertension	110 (56)	35 (43)	75 (66)	0.001
Chronic kidney disease (eGFR < 60)	111 (57)	61 (74)	50 (44)	1.8 × 10^−4^
Diabetes mellitus	64 (33)	36 (44)	28 (25)	0.024
Dyslipidemia	78 (40)	35 (43)	43 (38)	0.86
Ever smokers	104 (53)	52 (63)	52 (46)	0.011
Current smokers	24 (12)	9 (11)	15 (13)	0.78
Medications, *n* (%)				
ACE inhibitors/ARB	131 (67)	70 (85)	61 (54)	6.0 × 10^−7^
β-blockers	123 (63)	65 (79)	58 (51)	1.1 × 10^−4^
Calcium channel blockers	47 (24)	15 (18)	32 (28)	0.040
MRA	55 (28)	36 (44)	19 (17)	1.8 × 10^−5^
Diuretics	100 (51)	53 (65)	47 (41)	0.001
Statin	63 (32)	29 (35)	34 (30)	0.79
Physiological experiments				
RV5 + SV1 in ECG, mV	2.65 ± 1.61	2.19 ± 1.35	2.98 ± 1.70	0.001
LAD, mm	45.2 ± 8.3	46.5 ± 8.6	44.3 ± 7.9	0.072
IVST, mm	10.8 ± 3.7	8.8 ± 2.6	12.2 ± 3.8	2.2 × 10^−10^
PWT, mm	10.0 ± 2.5	8.8 ± 1.9	10.8 ± 2.5	1.3 × 10^−7^
LVDd, mm	53.1 ± 11.4	61.6 ± 11.6	47.0 ± 6.2	2.6 × 10^−21^
LVDs, mm	40.0 ± 14.9	53.1 ± 13.2	30.4 ± 6.3	2.6 × 10^−29^
LVMI, g/m^2^	133 ± 45	140 ± 43	127 ± 46	0.001
EF, %	51.4 ± 18.8	32.7 ± 11.9	64.2 ± 8.6	1.4 × 10^−31^
E wave, cm/s	83.4 ± 29.5	84.0 ± 30.7	82.9 ± 28.7	0.778
A wave, cm/s	64.0 ± 31.2	58.2 ± 30.3	68.7 ± 31.4	0.022
E to A ratio	1.73 ± 2.07	1.86 ± 1.28	1.63 ± 2.54	0.070
Deceleration time, ms	221 ± 83	205 ± 79	232 ± 85	0.022
E’ wave, cm/s	7.4 ± 3.0	6.6 ± 2.8	8.0 ± 3.1	1.5 × 10^−4^
E to E’ ratio	12.9 ± 7.8	14.9 ± 8.8	11.5 ± 6.7	0.001
Laboratory experiments				
Hemoglobin, g/dl	12.5 ± 2.2	12.5 ± 2.2	12.4 ± 2.2	0.93
Na, mEq/L	139.7 ± 3.7	139.0 ± 3.6	140.2 ± 3.7	0.009
K, mEq/L	4.39 ± 0.56	4.38 ± 0.55	4.40 ± 0.57	0.904
Cl, mEq/L	103.2 ± 8.6	102.7 ± 4.7	103.5 ± 10.5	0.012
Low-density lipoprotein, mg/dL	95.3 ± 32.7	93.0 ± 33.0	96.8 ± 32.6	0.14
High-density lipoprotein, mg/dL	55.2 ± 15.5	50.4 ± 12.4	58.3 ± 16.6	0.002
Triglyceride, mg/dL	130.0 ± 123.6	144.9 ± 175.0	120.0 ± 67.8	0.664
eGFR, ml/min/1.73 m^2^	49.8 ± 23.6	44.6 ± 24.9	53.5 ± 22.1	0.002
Cre, mg/dl	1.52 ± 1.60	1.87 ± 2.07	1.28 ± 1.11	1.5 × 10^−5^
HbA1c, %	6.20 ± 1.10	6.30 ± 1.00	6.12 ± 1.16	0.259
Fasting glucose, mg/dL	118.8 ± 38.4	120.3 ± 40.6	117.8 ± 37.0	0.508
U-Alb/gCre, mg/g	307 ± 1207	108 ± 283	392 ± 1430	0.958
Urine β2-microglobulin, μg/L	4611 ± 9874	2037 ± 3268	6508 ± 12,517	1.00

Values are mean ± SD or *n* (%). Abbreviations: BNP, brain natriuretic peptide; ECG, electrocardiography; EF, ejection fraction; eGFR, estimated glomerular filtration rate; HFpEF, heart failure with preserved ejection fraction; HFrEF, heart failure with reduced ejection fraction; IVST, interventricular septal wall thickness; LAD, left atrial dimension; LVDd, left ventricular end-diastolic dimension; LVDs, left ventricular end-systolic dimension; LVEF, left ventricular ejection fraction; LVMI, left ventricle mass index; PWT, posterior wall thickness; U-Alb/gCre, urine albumin to gram creatinine ratio.

**Table 2:** Univariate analysis and multivariate regression analysis of the ratio of iBNP to BNP in patients with HF.

Variables	Univariate		Multivariate	
	β	*p* Value	β	*p* Value
HFpEF	0.194	0.007	0.154	0.032
Age	0.175	0.014	0.167	0.025
Hemoglobin	0.167	0.020	0.203	0.006
Calcium channel blockers	0.153	0.032	0.138	0.049
Deceleration time	0.167	0.022	0.143	0.049
Male	0.067	0.351	0.029	0.692
Body mass index	0.058	0.418	0.024	0.740
Diabetes mellitus	−0.032	0.661	−0.007	0.925

Abbreviations: BNP, brain natriuretic peptide; HFpEF, heart failure with preserved ejection fraction; iBNP, serum-induced brain natriuretic peptide.

## Data Availability

Data supporting the reported results are available on request from the corresponding author.

## Abbreviations

BNP; brain natriuretic peptide; HF, heart failure; HFpEF, heart failure with preserved ejection fraction; HFrEF, heart failure with reduced ejection fraction; iBNP, serum-induced BNP. KI, knock-in; pBNP, promotor of BNP.
